# Determining how different ventilation shutdown plus methods change the electroencephalography, blood chemistry, corticosterone, and heat shock protein 70 of laying hens

**DOI:** 10.3389/fphys.2025.1534385

**Published:** 2025-03-21

**Authors:** Kari L. Harding, Emmillie Boot, Jackson O. Evans, Sanjay B. Shah, Ramon D. Malheiros, Kenneth E. Anderson

**Affiliations:** ^1^ Prestage Department of Poultry Science, North Carolina State University, Raleigh, NC, United States; ^2^ Biological and Agricultural Engineering, North Carolina State University, Raleigh, NC, United States

**Keywords:** depopulation, physiology, relative humidity, highly pathogenic avian influenza, laying hen

## Abstract

The poultry industry faces a major impediment in dealing with highly pathogenic avian influenza (HPAI). Large outbreaks have resulted in depletion of available resources needed for desired depopulation methods, leading to the need for alternative methods. This study was conducted to explore alternative ventilation shutdown procedures and how they affect laying hens throughout the process. Three treatments evaluated were ventilation shutdown plus heat (VSDH), ventilation shutdown plus heat and relative humidity (VSDHRh), and ventilation shutdown plus carbon dioxide (VSDCO_2_). There were two phases used: one phase was used to study treatment effects on the hens’ EEG responses from beginning to time of death and how laying hens behaved. Phase 2 examined how these treatments affected hen blood chemistry and HSP70 during the process. VSDCO_2_ had a significantly quicker time of death (P = 0.0003), and VSDH and VSDHRh were not different. There were no differences in pre- or post-corticosterone levels in Phase 1; however, there was a trend (P = 0.07) toward significance in the post corticosterone levels. Heat shock protein 70 (HSP70) levels were higher (P = 0.0001) in the VSDCO_2_ treatment, which could be due to the protein upregulation to prevent apoptosis. In Phase 2, VSDH corticosterone had a significantly greater treatment effect compared to VSDHRh and VSDCO_2_. corticosterone levels were significantly greater than those of VSDHRh. There were no significant treatment effects in Phase 2 for HSP70 expression; however, the sequence was significant, with the HSP70 being significantly greater at 75% to the average time of death than at 100% to the average time of death. Overall, VSDHRh could be a good alternative for the industry to use to rapidly depopulate laying hen facilities. However, more research on this treatment and more in-depth stress parameters measured needs to be conducted to fully determine how it affects laying hens.

## 1 Introduction

Highly pathogenic avian influenza (HPAI) is an infectious disease that has become a major concern for the poultry industry. Beginning in 2014 and carrying over into 2015, the poultry industry experienced a major outbreak of HPAI. This outbreak resulted in the loss of roughly 45 million birds, mostly laying hens or pullets (Greene, 2015). Currently an outbreak of HPAI that was first reported in 2022 and is ongoing has led to 96.91 million birds being depopulated. Of these flocks, 496 have been commercial and 655 have been backyard flocks (USDA, 2024). According to the American Veterinary Medical Association (AVMA), depopulation is defined as “methods by which large numbers of animals must be destroyed quickly and efficiently with as much consideration given to the welfare of animals as practicable, given extenuating circumstances” (AVMA, 2019). Current preferred depopulation methods for floor-reared poultry include use of water-based expanding foam, captive bolt guns, and water-based foam nozzles. Cage-housed laying hens have permitted methods such as CO_2_ kill carts, CO_2_ injection throughout the entire house, and partial-house gassing. The injection of CO_2_ for whole-house or partial-house is a preferred method for floor-reared poultry as well. Under constrained circumstances, some of the following methods are permitted for floor-reared poultry: (VSD) plus (hyperthermia), controlled demolition, and exsanguination, and for caged-house poultry, use of compressed air foam, captive bolt guns, and VSD+ are some of the permitted methods. While all of these methods are successful, the preferred methods are most often quicker methods of depopulation compared to the permitted methods for both housing systems. Ventilation shutdown alone is not permitted for either of these poultry housing types, and therefore was not used for this study. The challenge that was identified from the outbreaks was that multi-tiered housing systems such as conventional or colony cages and aviaries, along with high-rise houses, provided hindrances to some of the preferred depopulation methods. In both outbreaks, supplies, namely, CO_2_, were rapidly depleted or not available, resulting in an increase in the suffering of affected birds and leading to the potential for a greater spread of the virus. This led to the need for development of alternative depopulation methods to decrease the potential for a greater spread of the disease and to mitigate bird suffering and biosecurity risks. Eberle-Krish et al. (2018) evaluated ventilation shutdown plus CO_2_ (VSDCO_2_) and ventilation shutdown plus heat (VSDH) vs ventilation shutdown (VSD). They reported that VSDCO_2_ was more rapid than all other treatments, followed by VSDH, both with 100% mortality compared to VSD alone, which did not meet the 100% mortality standard. Other research studies have found that including high relative humidity levels with heat reduces the time it takes for total depopulation (Zhao et al., 2019). Another study looking at the addition of steam showed that all treatments using steam were significantly faster to observe the first hen death and complete mortality compared to ventilation shutdown plus heat alone (Mendoza and Williams, 2024). There is very limited research evaluating how laying hen electroencephalograms (EEGs) change throughout any of the VSD processes. One study evaluated the EEGs of laying hens during application of whole-house CO_2_. The results in individual birds indicated the changes between normal, transitional, suppressed, and isoelectric EEG phases. The results reported that out of ten laying hens, nine exhibited a decrease in EEG amplitude at each of the phases (Mckeegan et al., 2011). Another trial utilized EEGs to determine time to unconsciousness and brain death in broilers, layers, turkeys, and ducks. It was reported that turkeys reached time to brain death faster when water-based foam was used, compared to layers and ducks in which they reach brain death sooner with use of CO_2_ gas (Benson et al., 2012). While these studies have observed what one method looks like for laying hens, there are no known studies that have compared EEG signals of laying hens across treatments. There is currently no research comparing the different ventilation shutdown plus methods for their effects on laying hen blood physiology and behaviors either.

## 2 Materials and methods

All research procedures involving animals in these trials were approved by the North Carolina State University Institutional Animal Care and Use Committee (IACUC # 21-310). This study was conducted in the Bird Wing of the Prestage Department of Poultry Science at North Carolina State University. Throughout the study, all animals were monitored by veterinarians and animal welfare specialists employed by the university.

Both Phase 1 and Phase 2 experiments described below were conducted in four chambers that were housed in a windowless, temperature-controlled room. The chambers were made with Plexiglass® with fittings that allowed for CO_2_ sampling using external meters to collect data and for the injection of gas or humidity. The temperature and relative humidity for each chamber was recorded using Aranet sensors (Aranet, Riga, Latvia; model: SKU: TDSPT8U2; accuracy: ±0.3°C and ±2%). These sensors recorded every minute with the base placed approximately 4 cm (0.04 m) from the top of each chamber and the sensor reaching bird eye level when standing. Three sides, top, and bottom were covered with 2.5 cm of the closed cell insulation board, with only the front panel uncovered to all the hens to be observed. The chamber size was 1.68 ft. (0.51 m) × 1.68 ft. (0.51 m) × 1.68 ft. (0.51 m) equating to the space volume per hen in a typical caged layer facility. The room containing the chambers was maintained at 26°C to prevent heat loss to the environment. In each chamber, a 100-W incandescent light bulb built into the chamber was used as the heat source for the heat treatments described in Table 1. This allowed for temperatures to reach 40°C, which was chosen based on the Department of Environment, Food, and Rural Affairs (DEFRA, 2009). All chambers were equipped with raised 1 in. × 2 in. (0.305 m × 0.610 m) welded wire floors to mimic cages floors and to allow the hens to maintain posture. A total of three treatments were analyzed in both Phase 1 and Phase 2, and they included ventilation shutdown plus heat (VSDH), ventilation shutdown plus heat and relative humidity (VSDHRh), and ventilation shutdown plus CO_2_ (VSDCO_2_). Phase 1 had four replicates per treatment, totaling 12 white commercial laying hens that were approximately 69 weeks of age. These hens were maintained in the Bird Wing at North Carolina State University and were randomly assigned to the treatments. These hens were housed individually, in conventional cages with no additional treatments or procedures for at least 1 week prior to trial initiation. Treatment hens were removed, and blood was obtained from the brachial vein to obtain a baseline level of blood chemistry and corticosterone levels prior to treatment. Core body temperatures for each hen were evaluated using a SuperMeter® (Stamford, CT, United States; Model: HHM290) before and after treatment. The brain electrical output was measured in millivolts (mV) by using an electroencephalogram (EEG). Individual electrodes were insulated, except at the tips, and were attached to the pre-amplifier (AD Instruments, Colorado Springs, CO, United States), which transfers the EEG signals to a laptop-based recording that was continuously monitored. Prior to electrode placement, the birds were hobbled, which allowed them to stand and walk, but prevented them from scratching their heads and pulling out the electrodes. Hobbling was conducted using a rubber band that was placed on the shanks and above the dewclaws. The electrode tips were 32-gauge needles attached to the insulated wire and were inserted subcutaneously. Electrode colors are of three types: red, black, and green. The red electrode was placed on one side of the hen’s cranium, and the black electrode was placed on the other side. The green electrode, also known as the ground, was inserted between the wattles in the lower mandible near the neck. Each electrode tip was secured in its place on the head by using surgical adhesive by employing the catheter taping method. These three leads were then taped together and wound under the wing scapular joint, reducing the probability of the bird getting tangled up and pulling the leads out. Once electrodes were placed, the bird was placed into a chamber with the included treatment.

**TABLE 1:** Description of the ventilation shutdown alternative treatments for the trial.

Treatment	Treatment code	Description
Ventilation shutdown plus heat	VSDH	Ventilation is turned off, all inlets and exhausts are sealed, and heat is added
Ventilation shutdown plus heat and relative humidity	VDSHRh	Ventilation is turned off, air inlets and exhausts are sealed, and both heat and humidity are injected into the chambers with the target of ∼99% Rh
Ventilation shutdown plus carbon dioxide	VSDCO_2_	Ventilation is turned off, air inlets and exhausts are sealed, and CO_2_ is injected into the chamber to reach 30% concentration

The start time was determined at the point the chamber was sealed, and the EEG was plugged into the pre-amplifier. The EEG data were recorded at a standard frequency band of 10–50 Hertz (Hz) and sampled at 100 Hz/channel. The EEG was recorded until time of death was called by a veterinarian. Each hen’s behaviors were monitored throughout the treatment and recorded in real time every 2 min by a trained observer. To keep up with these 2-min checks, timers were utilized. Behaviors that were observed and their descriptors are described in Table 2 based on definitions by Hurnik et al. (1995). The CO_2_ concentrations were measured with two 0%–100% CO_2_ monitors (CO_2_ Meter, Inc., Ormond Beach, FL; model: CM-0003; accuracy: ±70 ppm ± 5% of measured value). These were also recorded on minute intervals as well. Once time of death (TOD) was called for each hen, they were removed from the treatment chamber, with blood being collected by severing the hepatic artery and collecting blood samples from the body cavity within 5 min of TOD. Blood was collected in BD Vacutainer tubes containing lithium heparin to prevent clotting. Approximately 0.1 mL of heparinized blood was then placed on the *i*-STAT® diagnostic system using CG8+ cartridges (Abbott Park, IL, United States; Model: 03P8825) for blood chemistry analysis. Birds were then necropsied with the brain being collected and placed into RNA*later*® (Thermo Fisher Scientific, Waltham, MA: Catalog #AM7021) solution. This tube was placed in a refrigerator at 4°C for 24 h and afterward moved to a −20° freezer.

**TABLE 2:** Description of the behaviors^a^ analyzed and reported every 2 min as hens underwent treatment.

Behavior	Description
Conscious	
Headshake	Rapid shaking or lateral movement of the head
Mandibulation	Repetitive tasting movement with the beak
Standing	Legs extended, fully upright
Wing flapping	A bout of continuous, rapid wing flapping
Crouch	Legs are folded under the bird with the body positioned on top
Unconscious	
Panting	Deeper than normal expiration through an open mouth generally accompanied by movements of the tongue and beak
Respiratory disruption	Deep, open beak breathing with prolonged inspiration or prolonged open beak gasping, or both, combined with difficulty inhaling
Loss of posture	Loss of balance or posture or both (lateral recumbency

^a^ Behaviors based on definitions by Hurnik et al. (1995).

Phase 2 of this trial specifically compared the three treatments with respect to physiological changes (blood chemistry and gene expression) over four time points. Ten ∼69-week-old hens were used for each treatment with two replications per treatment, totaling 30 hens. The TOD for the birds in Phase 1 was averaged for each treatment. The average TOD was then quartered, giving a total of four-time intervals, plus one baseline bird that never entered the chamber. The calculated time points to remove the birds are shown in Table 3. This allowed for the evaluation of the hens at the physiological progression over time. At each calculated removal time point, the hen was removed from the chamber, and her blood was collected for blood chemistry and corticosterone analyses within 60 s. The hen was restrained on its side and allowed to bleed via the brachial vein. Between 1.5 and 2 mL of the sample blood was collected using a 3-mL syringe and then transferred to a BD Vacutainer tube with lithium heparin. Then, a 0.1 mL sample of the heparinized blood was placed in the *i*-STAT® diagnostic system using CG8+ cartridges for blood chemistry analysis. The remaining blood was gently shaken to prevent clotting. The blood was then centrifuged and the plasma supernatant collected and frozen at −20°C for future corticosterone analysis. The hen was then euthanized by a trained individual via cervical dislocation, and the brain tissue was collected and placed in RNA*later*® and refrigerated at 4°C for 24 h then moved to a −20°C freezer for later gene expression analysis.

**TABLE 3:** Calculated sampling times in minutes of removal times to determine changes in blood chemistry and HSP70^c^ over time.

Sequence^a^	0	25%	50%	75%	100%
Treatment^b^	Minutes				
VSDH	0	14	28	42	56
VSDHRh	0	12	24	36	48
VSDCO_2_	0	6	12	18	24

^a^ Sequence is baselines = 0, 25 is 25% to average TOD, 50 is 50% to average TOD, 75 is 75% to average TOD, and 100 is average TOD.

^b^ VSDH, ventilation shutdown plus heat; VSDHRh, ventilation shutdown plus heat and relative humidity; VSDCO_2_ = ventilation shutdown plus carbon dioxide.

^c^ HSP70, heat shock protein 70.

Plasma corticosterone levels for Phase 1 and Phase 2 were collected by using an ELISA corticosterone kit following the manufacturer’s guidelines. Corticosterone concentrations were determined by a standard curve as nanograms of corticosterone per milliliter of plasma with each sample run in duplicates. The kit was validated using known standards for each plate run, plotting the values, and adding a best fit line. Values were deemed acceptable with an R^2^ value of ≥0.90 (Cayman Chemical Company, Item: 501320, Ann Arbor MI, United States). For brain samples from Phase 1 and Phase 2, an approximately 1-cm sample of the brain was taken to isolate RNA. These samples were placed in homogenization vials with screw caps, and 1 mL of TRI Reagent™ solution was added to each sample. Each sample was then homogenized using a bead-beater for 20 s and then allowed to incubate at room temperature for 5 min. Then, 200 µL of chloroform was administered to each sample and then vortexed for approximately 10 s. The sample was allowed to sit at room temperature for 2 min and then centrifuged for 20 min at 13,000 × g. Completion of the RNA isolation was performed using an RNeasy kit (QIAGEN, Hilden Germany) according to the manufacturer’s guidelines. RNA levels were quantified using the NanoDrop 2000 (Thermo Fisher Scientific-Waltham, MA) to ensure the correct RNA dilutions were obtained. The ideal RNA concentration was between 100 and 1,000 ng/μL, and these were utilized due to ease of pipetting in downstream applications. The RNA was then transcribed into cDNA for qPCR by utilizing a high-capacity cDNA synthesis kit (Applied Biosystems Waltham, Massachusetts, United States). The qPCR was completed on each sample in triplicate. All wells contained 2.5 ng of samples, 500 nM of gene specific forward and reverse primers (Table 4), and 2X power SYBR green master mix (Applied Biosystems Waltham, Massachusetts, United States), and RNase-Free H_2_O was added to finalize the volume to 20 µL. The qPCR was performed on the Applied Biosystems StepOnePlus real-time PCR system. Results were normalized to the expression of the basic housekeeping gene, beta actin. The reciprocal was taken for ease of interpretation. The formula used for this is as follows: 1/(target gene cycle threshold/beta actin cycle threshold). All data were then converted to the reciprocal cycle thresholds or CT^−1^. ΔΔCT was then calculated by first averaging each treatment CT^−1,^ and VSDH was set to be the control. The average for VSDH was then subtracted from each CT^−1^ to obtain the ΔCT. Because VSDH was used as the control, the ΔΔCT values are the same as the ΔCT values. Each of these values were put into the following equation: 2^−ΔΔCT^.

**TABLE 4:** Genes and their genetic sequences (forward and reverse) utilized for gene expression.

Gene	Primer	Directional sequence	Sequence
Beta actin	b-Actin	Forward	GTCCACCTTCCAGCAGATGT
		Reverse	ATAAAGCCATGCCAATCTCG
Heat shock protein	HSP70	Forward	GCGGAGCGAGTGGCTGACTG
		Reverse	CGGTTCCCCTGGTCGTTGGC

## 3 Statistical analysis

A one-way ANOVA was used to evaluate the treatment differences in Phase 1, and a two-way ANOVA was used to evaluate if there were any treatment, sequence, or interaction effects between the two in Phase 2. Significant differences were accepted with α ≤ 0.05, and if there were any differences observed, a Tukey’s HSD was utilized for pairwise comparisons. The EEG data were transformed by taking the absolute value of the integral, allowing for the mitigation of the baseline noise, which was relative to the baseline at each 10-s interval, which was conducted using the following equation:

$$\left|\int F|\left(t\right)|dt\right|$$

A hyperbolic arcsine function was used on each value to emphasize the lower millivolt (mV) readings. These transformed EEG data were then analyzed with GLM with full factorial effects of CO_2_, heat, and heat and humidity fit to each of several response variables. The transformed EEG data used the integral area under the curve that was calculated using the trapezoid method using an NPARM analysis. Behavior data were summarized as a frequency of behaviors that the hens performed as conscious (voluntary) or unconscious (involuntary) behaviors, overlaid with summarized EEG brain activity over the same time intervals. The correlation analysis examined the relationship between the VSDH, VSDHRh, VSDCO2, laying hen EEGs and the behavior profiles. Electroencephalogram activity and conscious and unconscious behaviors was analyzed using Pearson Linear Correlation Analysis in SAS JMP-PRO® 12.2.0 (SAS Institute, 1989).

## 4 Results and discussion

Table 5 shows the starting and ending chamber temperatures along with relative humidity for each treatment as well as start and end CO_2_ levels. There were no significant differences between starting relative humidity or starting CO_2_ values of the chamber. There was a significant difference in the start chamber temperature, with the VSDHRh being significantly higher than the starting chamber temperature in the VSDCO_2_ due to the heating of the outside room for both VSDH and VSDHRh and not the VSDCO_2_ treatment. There was a significantly lower end chamber temperature for VSDCO_2_ compared to the other two treatments. This is because this is the only treatment that does not have heat supplemented. The ending relative humidity percentage was significantly greater in the VSDHRh compared to the other two treatments due to addition of humidity. The ending CO_2_ levels were significantly greater in VSDCO_2,_ whereas the other two treatments were not different from one another. Table 6 depicts that while there were no significant differences in the pre-core body temperature of hens among the treatments, there was a significantly lower post-core body temperature in the VSDCO_2_. This again, is due to there being no additional heat being supplemented to this treatment, and the mode of death was hypoxia rather than hyperthermia.

**TABLE 5:** Start and end temperature, relative humidity, and CO_2_ levels for each treatment.

Treatment^a^	Start chamber temperature	Start chamber relative humidity	Start CO_2_	End chamber temperature	End chamber relative humidity	End CO_2_
	(°C)	(%)	(ppm)	(°C)	(%)	(ppm)
VSDH	31.59^ab^	44.35	0.19	44.05^a^	61.63^b^	1.79^b^
VSDHRh	32.40^a^	45.00	0.17	43.63^a^	91.08^a^	1.28^b^
VSDCO_2_	28.05^b^	52.88	0.24	29.49^b^	64.70^b^	22.73^a^
Std. Dev	1.60	1.82	0.05	0.46	3.92	0.52
P-value	0.0249	0.2002	0.504	<0.0001	<0.0001	<0.0001

^a^ VSDH, ventilation shutdown plus heat; VSDHRh, ventilation shutdown plus heat and relative humidity; VSDCO_2_, ventilation shutdown plus carbon dioxide.

**TABLE 6:** Pre- and post-core body temperatures and time of death of hens subjected to each treatment.

Treatment^a^	Hen body weight	Core body temperature (°C)		Time of death
	(kg)	Pre (°C)	Post (°C)	(Minutes)
VSDH	1.81	40.20	45.33^a^	54.50^a^
VSDHRh	1.88	40.98	45.48^a^	45.75^a^
VSDCO_2_	1.72	39.43	40.98^b^	24.50^b^
Std. Dev	0.09	0.93	1.37	1.00
P*-*value	0.4270	0.0961	0.0003	0.0003

^a^ VSDH, ventilation shutdown plus heat; VSDHRh, ventilation shutdown plus heat and relative humidity; VSDCO_2_, ventilation shutdown plus carbon dioxide.

**FIGURE 1:**
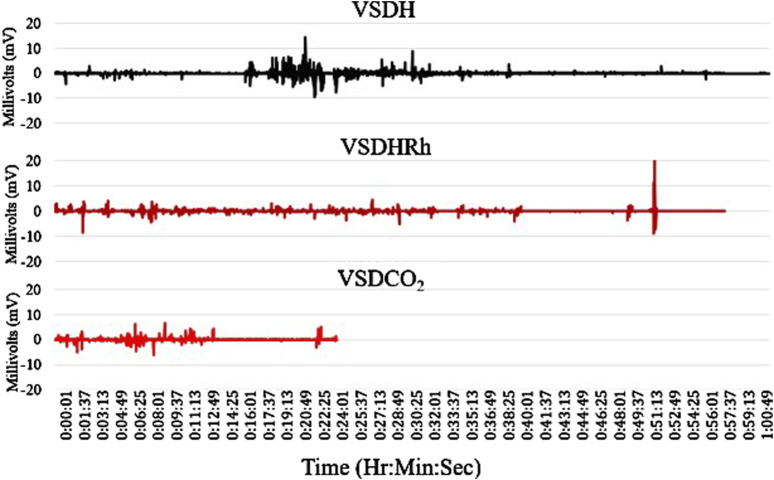
Composite electroencephalograms (EEGs) of the four birds per each treatment for VSDH, VSDHRh, and VSDCO2 from initiation to time of death (TOD). VSDH, ventilation shutdown plus heat; VSDHRh, ventilation shutdown plus heat and relative humidity; VSDCO_2_, ventilation shutdown plus carbon dioxide.

**FIGURE 2:**
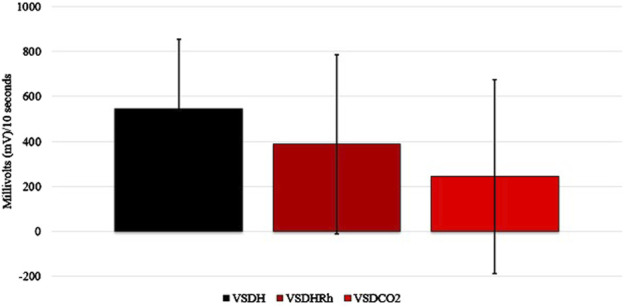
Integral area under the electroencephalogram (EEG) graph calculated of the transformed composite EEGs for VSDH, VSDHRh, and VSDCO_2_ through TOD using the trapezoid method. VSDH, ventilation shutdown plus heat; VSDHRh, ventilation shutdown plus heat and relative humidity; VSDCO_2_, ventilation shutdown plus carbon dioxide.

**FIGURE 3:**
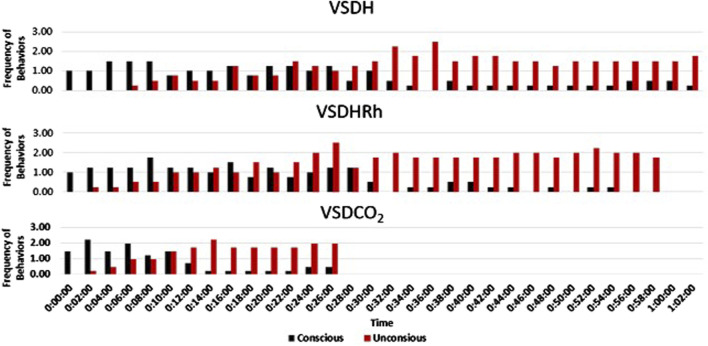
Frequency of conscious (voluntary) and unconscious (involuntary) behavioral responses for VSDH, VSDHRh, and VSDCO_2_ through to time of death. VSDH, ventilation shutdown plus heat; VSDHRh, ventilation shutdown plus heat and relative humidity; VSDCO_2_, ventilation shutdown plus carbon dioxide. Unconscious behaviors were determined when the electroencephalogram (EEG) readings were below 0.01 millivolts.

**FIGURE 4:**
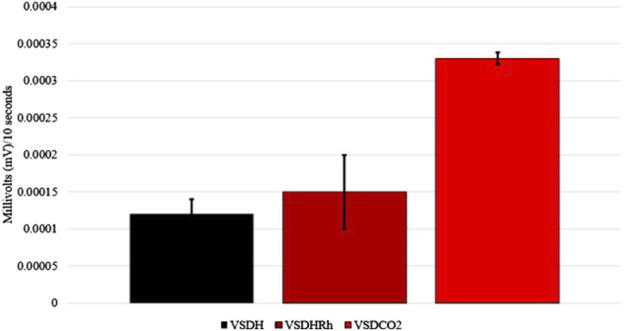
Slopes of the transformed electroencephalogram (EEG) readings of laying hens undergoing different methods of ventilation shutdown. VSDH, ventilation shutdown plus heat; VSDHRh, ventilation shutdown plus heat and relative humidity; VSDCO_2_, ventilation shutdown plus carbon dioxide.

The TOD in minutes is reported in Table 6, and the VSDCO_2_ treatment has a significantly shorter time of death at 24.50 min than the other two treatments analyzed. This was expected based on previous studies (Eberle-Krish et al., 2018). Results from the composite EEG for all treatments are shown in Figure 1. These results indicate that hens did go unconscious in the later stages of these methods, with sporadic spikes in the EEG mV intensity. This agrees with those results found by Mckeegan et al. (2011) which observed relatively consistent changes over time within the laying hen EEGs. This study also reported that the EEG signal was heavily affected soon after CO_2_ was injected, which was observed in this study. Figure 2 depicts the integral area under the electroencephalographic (EEG) composite graph for VSDH, VSDHRh, and VSDCO_2_ to TOD using the trapezoidal method. The area under the graph of these transformed EEGs was not different among the three treatments. This was unexpected because the TOD for the VSDCO_2_ treatment was significantly shorter. These results could be because the brainwave activity was variable and relatively high throughout the initial time for all the methods. Figure 3 depicts the frequency of voluntary and involuntary behavioral responses from beginning to time of death. There were no significant differences between the behaviors of the birds undergoing their respective treatments. There were no significant differences in the strength of the EEGs compared to all other treatments, as shown in Table 7. This indicates that even though the EEGs shown in Figure 1 had different patterns of mV strength, there was no difference in the percentages of the mV strengths between the methods. This is supported by the area under the EEG graphs shown in Figure 2, which indicates there are no differences between the depopulation methods compared in this work. There were significant shifts in conscious and unconscious behavior observed, as shown in Figure 3. At the midpoint of each depopulation method, the shift from conscious to unconscious behaviors was dramatic and consistent. This appears to correspond with Wang et al. (2016) observations, where a decline in neuron function in hyperthermia conditions above normal core body temperature was observed. Table 8 illustrates that based on the behavior observations as they relate to the EEG wave strength, there is a poor correlation between hen behaviors and EEG wave strength. VSDH was significant for both conscious and unconscious behaviors, as shown in Table 8. Figure 4 depicts the slope of the transformed EEG reading for each treatment. There was no significant difference between them, showing that there was no difference in magnitude over the course of each treatment when compared to one another.

**TABLE 7:** Effect of ventilation shutdown treatment groups on strength of the electroencephalographic waves (mV).

Treatment^a^	Percent EEG time within each mV range^b^			
	0–0.01 mV	0.01–0.03 mV	0.03–0.05 mV	>0.05 mV
	(%)	(%)	(%)	(%)
VSDH	34.76	16.65	4.96	43.63
VSDHRh	45.72	14.69	5.02	34.57
VSDCO_2_	49.68	6.09	5.94	38.28
Std. Dev	17.92	2.90	2.27	17.45
P*-*value	0.8333	0.0655	0.9426	0.9347

^a^ VSDH, ventilation shutdown plus heat; VSDHRh, ventilation shutdown plus heat and relative humidity; VSDCO_2_, ventilation shutdown plus carbon dioxide.

^b^ Percent EEG time within each mV range explain how long birds were unconscious (0–0.01 mV) and when they were experiencing higher neural activity, which are greater in >0.05 mV and how they vary between the two levels.

**TABLE 8:** Pearson linear correlation coefficient associated with behavior observations as they relate to the EEG^d^ strength.

Treatment^a^	Conscious^b^	Confidence interval			Behavior
	>0.01 mV	Lower 95%	Upper 95%	P-value	N =
VSDH	0.13	2.32	2.35	0.0001	112
VSDHRh	0.00	−0.04	−0.05	0.91	95
VSDCO_2_	0.05	0.78	0.75	0.11	52

Treatment	Unconscious^c^	Confidence interval			Behavior
	<0.01 mV	Lower 95%	Upper 95%	P-value	N =
VSDH	0.01	2.32	2.35	0.0002	112
VSDHRh	0.00	−0.04	−0.05	0.93	95
VSDCO_2_	0.04	0.78	0.75	0.18	52

^a^ VSDH, ventilation shutdown plus heat; VSDHRh, ventilation shutdown plus heat and relative humidity; VSDCO_2_, ventilation shutdown plus carbon dioxide.

^b^ Conscious behaviors were defined as voluntary behaviors.

^c^ Unconscious behaviors were defined as involuntary and were determined when the EEG readings dropped below 0.01 mV.

^d^ EEG, electroencephalogram.

**FIGURE 5:**
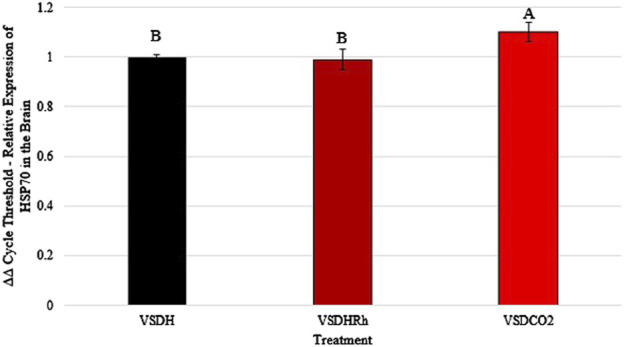
*Heat shock protein 70 (HSP70) levels of laying hens exposed to different methods of ventilation shutdown. VSDH, ventilation shutdown plus heat; VSDHRh, ventilation shutdown plus heat and relative humidity; VSDCO_2_, ventilation shutdown plus carbon dioxide. A, B — different uppercase letters denote significant differences P < 0.05. *Depicts parameters with a power of analysis of >0.80.

Heat shock protein 70 (HSP70) levels were significantly upregulated in the VSDCO_2_ treatment compared to the other two treatments (Figure 5). HSP70 ensures correct protein folding and prevents apoptosis. This gene is heavily upregulated during stress but can be upregulated to make adjustments biologically (Hassan et al., 2019). The elevated levels observed in the VSDCO_2_ treatment could have just been a biological adjustment or could have been a response to the reaction to the CO_2_ injection, which is an irritant that could cause bird stress. However, more research should be conducted on this to pinpoint exactly why this occurred. Table 9 shows the baseline blood chemistry parameters for each treatment with only significant differences in sodium, potassium, and ionized calcium. This could be due to the stress of handling and new environment. No blood chemistry parameters in Phase 1 were significantly different among the treatments, as shown in Table 10. Table 11 depicts the corticosterone levels of hens subjected to different treatments before they entered the chamber, and after TOD was called and they were removed from the chamber. There were no significant differences observed between treatments for either pre or post treatment; however, there was a trend (P = 0.07) for laying hen corticosterone after chamber removal; therefore, there could have potentially been differences observed with a larger sample size.

**TABLE 9:** Comparison of baseline blood chemistry levels in laying hens before undergoing treatment.

Treatment^a^	VSDH	VSDHRh	VSDCO_2_	Std. Dev	P-value
pH	7.44	7.40	7.43	0.09	0.5151
pCO_2_(mmHg)	32.70	36.45	32.70	6.21	0.1297
pO_2_(mmHg)	59.00	71.60	63.24	22.09	0.3450
BEecf (mmol/L)	−2.13	−2.10	−3.40	3.17	0.5929
HCO_3_(mmol/L)	22.05	22.45	20.93	2.39	0.3349
TCO_2_(mmol/L)	23.13	23.50	22.00	2.31	0.3546
sO_2_(%)	90.63	91.40	92.61	1.58	0.4892
Na (mmol/L)	137.13^c^	142.80^b^	147.90^a^	1.60	<0.0001
K (mmol/L)	4.15^b^	4.92^a^	3.68^b^	0.40	<0.0001
iCa (mmol/L)	2.44^a^	1.54^b^	1.48^b^	0.13	<0.0001
Glucose (mg/dL)	231.00	217.70	221.60	8.60	0.1758
Hct (% PCV)	20.88^b^	23.90^b^	23.90^b^	1.66	0.0098
Hb (g/dL)	7.10^b^	8.12^a^	8.13^a^	0.56	0.0097

^a^ VSDH, ventilation shutdown plus heat; VSDHRh, ventilation shutdown plus heat and relative humidity; VSDCO_2_, ventilation shutdown plus carbon dioxide.

pCO_2_, partial pressure of CO_2_; pO_2_, partial pressure of O_2_; BEecf, base excess in extracellular fluid; HCO_3_, bicarbonate; TCO_2_, total CO_2_.

**TABLE 10:** Changes in laying hen blood chemistry when depopulated using VSDH, VSDHRh, or VSDCO_2_.

Treatment^a^	VSDH	VSDHRh	VSDCO_2_	Std. Dev	P-value
pH	7.12	7.20	7.18	0.31	0.89
pCO_2_ (mmHg)	61.70	45.55	75.25	39.73	0.50
pO_2_ (mmHg)	44.50	62.75	54.75	11.18	0.48
BEecf (mmol/L)	−11.50	−11.50	−4.67	5.67	0.10
HCO_3_ (mmol/L)	17.95	16.40	21.77	2.24	0.13
TCO_2_ (mmol/L)	20.00	17.75	23.00	1.73	0.23
sO_2_ (%)	63.25	73.50	84.00	11.53	0.33
Na (mmol/L)	142.25	139.50	142.75	2.50	0.47
K (mmol/L)	8.75	9.00	6.88	2.45	0.13
iCa (mmol/L)	1.56	1.80	1.57	0.20	0.63
Glucose (mg/dL)	262.75	324.00	265.75	54.54	0.21
Hct (% PCV)	22.50	22.50	24.25	0.96	0.65
Hb (g/dL)	7.65	7.65	8.25	0.33	0.66

^a^ VSDH, ventilation shutdown plus heat; VSDHRh, ventilation shutdown plus heat and relative humidity; VSDCO_2_, ventilation shutdown plus carbon dioxide.

pCO_2_, partial pressure of CO_2_; pO_2_, partial pressure of O_2_; BEecf, base excess in extracellular fluid; HCO_3_, bicarbonate; TCO_2_, total CO_2_.

**TABLE 11:** Changes in laying hen corticosterone by treatment before using VSDH, VSDHRh, or VSDCO_2_ and after.

	Treatment^a^	VSDH	VSDHRh	VSDCO_2_	Std. Dev	P-value
Pre	Corticosterone (ng/mL)	0.06	0.06	0.05	0.01	0.64
Post		0.11	0.10	0.12	0.001	0.07

^a^ VSDH, ventilation shutdown plus heat; VSDHRh, ventilation shutdown plus heat and relative humidity; VSDCO_2_, ventilation shutdown plus carbon dioxide.

Phase 2 looked at how these treatments affected the hens over time by removing the hens at specific time points. Due to the small sample size in this phase, a power of analysis was conducted with parameters with a power greater than 0.80 being labeled. There were no significant treatment or interaction effects with the HSP70 levels for Phase 2 as shown in Table 12. There was, however, a significant sequence effect. The 75% to average TOD was significantly greater than that of the 100% to average TOD. This was thought to be due to the HSP70 being overwhelmed with the misfolded proteins and apoptosis, which resulted in the downregulation of the HSP70 gene at the 100% average time of death. Table 13 depicts the treatment, sequence, and interaction of each on laying hen corticosterone before and after the trial. There were no treatment, sequence, or interaction effects on the pre-corticosterone levels. There also were no sequence or interaction effects on the post-corticosterone levels. A significant treatment effect was observed with VSDH having greater levels at 0.16 ng/mL than all treatments, with VSDCO_2_ at 0.12 ng/mL being significantly greater than VSDHRh at 0.06 ng/mL. The higher levels of corticosterone in VSDH align with higher levels that are observed in heat stress when compared to hens that are not (Li et al., 2020). Tables 14, 15 depict the baseline blood chemistry for Phase 2 laying hens before they were subjected to their respective treatments. The effects of treatment, sequence, and the interaction as it pertains to blood chemistry parameters from Phase 2 are shown in Tables 16, 17. There is currently no research on how ventilation shutdown plus (hyperthermia) or VSDCO_2_ affects blood chemistry. There were no treatment or sequence effects observed for pH, pCO_2_, pO_2_, BEecf, HCO_3_, TCO_2_, Na, K, iCa, and Hct. Some heat stress studies have reported no changes in blood pH (Barret et al., 2019). Other studies reported that they did see increases in blood pH (Koelkebeck and Odom, 1994). There were, however, sequence effects for sO_2,_ which dropped significantly at 25% to average TOD; however, they recovered and did not drop again. This could be due to potential increased respiration due to a new environment and the noises at the beginning of the trial. Glucose had a significant sequence effect as well, with both 75% and 100% to average TOD calculated were significantly greater compared to that of the baseline. There were no differences between the other two time points. Koelkebeck and Odom, 1995 also reported there was no change in plasma glucose when laying hens were subjected to heat stress and high CO_2_ exposure. Other studies reported that when increasing the environmental temperature for chicks to 40°C for 2 h resulted in no significant impact on blood glucose levels (Bogin et al., 1981). This agrees with our findings of no significant changes in the blood glucose levels when analyzing treatment effects. The significant increase in glucose for the final two points in the sequence could be due to the body increasing energy availability during the treatments overall. There were no significant interaction effects in either Na, glucose, Hct, sO_2_, pO_2_, HCO_3_, or TCO_2_. There was a significant interaction in pH with VSDCO_2_ becoming significantly lower with the 100% TOD having a pH of 7.04. This was expected due to the acidic environment inhalation of CO_2_ creates in blood. In turn, this resulted in a significantly higher pCO_2_ level in the blood stream at 88.90, which was higher than both 75% and 100% to average TOD in VSDHRh and higher than 75% average TOD in VSDH. The BEecf parameter was significantly lower in the 100% average TOD for VSDCO_2_ compared to the final two sequence points for both VSDH and VSDHRh, most likely due to the increased acidic environment caused by the CO_2_. There was a significantly greater amount of K in the VSDCO_2_ treatment at 50% and 100% to average TOD compared to all other treatments. This could possibly be due to the K trying to balance the body’s acidic environment. There was also an interaction for iCa with VSDHRh having significantly lower levels at 100% average TOD than all other treatment sequences, except for VSDHRh 75% average TOD. This potentially could be due to increased panting by the birds, leading to water loss.

**TABLE 12:** Treatment, sequence, and their interaction effects on laying hen HSP70 levels.

Treatment^b^		ΔΔCT
VSDH		1.00 ± 0.03
VSDHRh		1.00 ± 0.06
VSDCO_2_		1.04 ± 0.06
P-value		0.1274
Sequence^ac^		
0%		1.00 ± 0.02^ab^
25%		1.00 ± 0.004^ab^
50%		1.01 ± 0.02^ab^
75%		1.07 + 0.08^a^
100%		0.99 ± 0.99^b^
P-value		0.0378
TrtXSequence^a^		
VSDH	0%	1.00
	25%	1.00
	50%	1.00
	75%	1.00
	100%	1.00
VSDHRh	0%	1.01
	25%	1.01
	50%	1.00
	75%	1.06
	100%	0.93
VSDCO_2_	0%	0.98
	25%	1.00
	50%	1.02
	75%	1.15
	100%	1.06
P-value		0.1324

^a^ Sequence is baseline = 0, 25 is 25% to average TOD, 50 is 50% to average TOD, 75 is 75% to average TOD, and 100 is average TOD.

^b^ VSDH, ventilation shutdown plus heat; VSDHRh, ventilation shutdown plus heat and relative humidity; VSDCO_2_, ventilation shutdown plus carbon dioxide.

^c^ Depicts parameters with a power of analysis of >0.80.

**TABLE 13:** Effects of treatment, sequence, and their interaction on laying hen corticosterone pre and post treatment.

Treatment^bc^		Corticosterone (ng/mL)	
			Pre^c^	Post^c^
VSDH		0.27 ± 0.05^a-^	0.16 ± 0.02^a^
VSDHRh		0.03 ± 0.01^b^	0.06 ± 0.02^c^
VSDCO_2_		0.10 ± 0.08^ab^	0.12 ± 0.04^b^
P-value		<0.0001	<0.0001
Sequence^a^			
0%		0.10 ± 0.09	0.10 ± 0.09
25%		0.11 ± 0.10	0.10 ± 0.05
50%		0.17 ± 0.15	0.12 ± 0.04
75%		0.16 ± 0.14	0.12 ± 0.04
100%		0.12 ± 0.12	0.13 ± 0.03
P-value		0.1546	0.3508
TrtXSequence^a^			
VSDH	0%	0.20 ± 0.02	0.20 ± 0.02
	25%	0.24 ± 0.01	0.15 ± 0.003
	50%	0.31 ± 0.06	0.15 ± 0.003
	75%	0.32 ± 0.01	0.14 ± 0.0005
	100%	0.28 ± 0.01	0.15 ± 0.004
VSDHRh	0%	0.03 ± 0.001	0.03 ± 0.002
	25%	0.04 ± 0.02	0.05 ± 0.004
	50%	0.03 ± 0.02	0.06 ± 0.005
	75%	0.03 ± 0.02	0.06 ± 0.001
	100%	0.05 ± 0.008	0.09 ± 0.03
VSDCO_2_	0%	0.08 ± 0.08	0.08 ± 0.08
	25%	0.06 ± 0.02	0.09 ± 0.05
	50%	0.16 ± 0.15	0.14 ± 0.002
	75%	0.14 ± 0.08	0.14 ± 0.01
100%	0.04 ± 0.02	0.15 ± 0.003
P-value		0.5077	0.0618

^a^ Sequence is baseline = 0, 25 is 25% to average TOD, 50 is 50% to average TOD, 75 is 75% to average TOD, and 100 is average TOD.

^b^ VSDH, ventilation shutdown plus heat; VSDHRh, ventilation shutdown plus heat and relative humidity; VSDCO_2_, ventilation shutdown plus carbon dioxide.

^c^ Depicts parameters with a power of analysis of >0.80.

**TABLE 14:** Baseline blood chemistry values of laying hens before undergoing treatment.

Trt^b^	Seq^a^ (%)	pH	pCO_2_ (mmHg)	pO_2_ ^c^ (mmHg)	BEecf (mmol/L)	HCO_3_ (mmol/L)	TCO_2_ (mmol/L)
VSDH		7.34 ± 0.08	48.20 ± 9.38	63.80 ± 9.06	0.10 ± 2.63	25.82 ± 2.18	27.40 ± 2.36
VSDHRh		7.40 ± 0.05	36.30 ± 4.86	64.21 ± 14.33	−2.20 ± 2.82	22.72 ± 2.31	23.80 ± 2.20
VSDCO_2_		7.39 ± 0.05	42.32 ± 5.46	54.70 ± 20.62	0.90 ± 3.53	25.76 ± 3.16	26.80 ± 3.20
P-value		0.3836	0.1461	0.4517	0.3743	0.2151	0.1773
Sequence^a^							
0%		7.43 ± 0.05	37.73 ± 3.98	92.83 ± 41.13	0.67 ± 2.53	25.02 ± 1.93	26.17 ± 2.00
25%		7.33 ± 0.06	43.68 ± 8.22	88.17 ± 36.63	−3.50 ± 2.94	22.63 ± 2.83	23.83 ± 2.93
50%		7.36 ± 0.04	45.40 ± 8.22	98.00 ± 37.95	−0.33 ± 3.67	25.30 ± 3.75	26.67 ± 4.04
75%		7.38 ± 0.10	44.27 ± 12.71	78.00 ± 24.17	0.83 ± 2.93	26.00 ± 3.11	27.33 ± 3.41
100%		7.40 ± 0.06	40.28 ± 6.17	74.00 ± 22.59	0.33 ± 3.20	24.88 ± 2.43	26.00 ± 2.43
P-value		0.5055	0.7820	0.6357	0.4174	0.5623	0.5512
TrtXSequence							
VSDH	0	7.37 ± 0.004	40.20 ± 3.57	62.50 ± 2.12	−2.00 ± 3.11	23.35 ± 0.21	24.50 ± 0.71
	25	7.29 ± 0.01	51.55 ± 6.15	66.50 ± 2.12	−1.50 ± 3.53	24.95 ± 3,075	26.50 ± 3.54
	50	7.32 ± 0.09	52.60 ± 9.33	56.00 ± 4.24	1.00 ± 1.41	27.20 ± 0.14	29.00 ± 0.71
	75	7.32 ± 0.11	53.80 ± 16.69	65.50 ± 13.44	1.00 ± 2.69	27.15 ± 1.91	29.00 ± 2.83
	100	7.40 ± 0.14	42.85 ± 11.24	68.50 ± 20.51	2.00 ± 4.24	26.45 ± 1.91	28.00 ± 1.41
VSDHRh	0	7.47 ± 0.09	33.60 ± 7.35	63.21 ± 37.38	1.00 ± 2.12	24.60 ± 0.35	26.00 ± 0.71
	25	7.35 ± 0.04	34.20 ± 2.83	61.33 ± 8.49	−2.00 ± 4.95	19.00 ± 3.89	20.00 ± 4.24
	50	7.37 ± 0.03	34.80 ± 2.26	59.56 ± 33.44	−1.50 ± 2.45	20.20 ± 0.21	21.00 ± 0.46
	75	7.43 ± 0.08	37.30 ± 7.28	67.98 ± 40.31	2.50 ± 2.97	24.70 ± 1.27	26.00 ± 1.41
	100	7.40 ± 0.03	41.60 ± 4.10	64.01 ± 16.97	1.50 ± 1.54	25.10 ± 0.57	26.00 ± 0.71
VSDCO_2_	0	7.45 ± 0.03	39.40 ± 3.68	60.00 ± 12.73	3.00 ± 4.95	27.10 ± 4.17	28.00 ± 4.24
	25	7.33 ± 0.07	45.30 ± 7.36	59.00 ± 1.41	2.00 ± 1.41	23.95 ± 0.21	25.00 ± 1.31
	50	7.37 ± 0.02	48.80 ± 8.13	58.32 ± 37.48	3.00 ± 2.83	28.50 ± 3.61	30.00 ± 4.24
	75	7.40 ± 0.07	41.70 ± 2.26	53.50 ± 0.71	1.50 ± 6.36	26.15 ± 5.59	27.00 ± 5.66
	100	7.41 ± 0.01	36.40 ± 0.49	53.00 ± 38.18	−1.00 ± 2.82	23.10 ± 1.84	24.00 ± 1.41
P-value		0.9975	0.7820	0.1288	0.7682	0.6596	0.5897

^a^ Seq is baseline = 0, 25 is 25% to average TOD, 50 is 50% to average TOD, 75 is 75% to average TOD, and 100 is average TOD.

^b^ VSDH, ventilation shutdown plus heat; VSDHRh, ventilation shutdown plus heat and relative humidity; VSDCO_2_, ventilation shutdown plus carbon dioxide.

^c^ Depicts parameters with a power of analysis of >0.80.

pCO_2_, partial pressure of CO_2_; pO_2_, partial pressure of O_2_; BEecf, base excess in extracellular fluid; HCO_3_, bicarbonate; TCO_2_, total CO_2_.

**TABLE 15:** Baseline blood chemistry levels of laying hens before undergoing treatments.

Trt^c^		Na (mmol/L)	K^b^ (mmol/L)	iCa^b^ (mmol/L)	Glucose (mg/dL)	Hct (% PCV)	sO_2_ ^b^ (%)
VSDH		132.90 ± 4.67	4.99 ± 0.97	2.37 ± 0.29	232.00 ± 3.14	20.50 ± 0.93	89.40 ± 3.70^b^
VSDHRh		143.20 ± 20.57	4.44 ± 0.53	2.50 ± 0.18	248.60 ± 14.56	29.20 ± 10.30	99.20 ± 2.20^a^
VSDCO_2_		130.50 ± 14.06	5.03 ± 0.66	2.50 ± 0.21	233.10 ± 11.46	20.00 ± 6.45	85.40 ± 7.86^b^
P-value		0.0949	0.5350	0.5931	0.0645	0.0750	0.0035
Seq^a^							
0%		130.00 ± 12.92	4.80 ± 0.40	2.50 ± 0.26	238.33 ± 5.89	18.67 ± 3.02	95.17 ± 5.05
25%		143.33 ± 16.76	4.67 ± 0.31	2.40 ± 0.25	248.00 ± 18.07	28.67 ± 9.33	92.33 ± 5.43
50%		137.00 ± 16.08	4.93 ± 0.44	2.50 ± 0.14	251.00 ± 14.29	24.67 ± 8.59	85.00 ± 11.08
75%		136.83 ± 18.94	5.07 ± 1.63	2.38 ± 0.31	224.33 ± 16.77	25.67 ± 7.11	92.00 ± 2.12
100%		130.50 ± 8.08	4.63 ± 0.27	2.50 ± 0.17	227.83 ± 15.46	18.50 ± 2.35	92.17 ± 5.99
P-value		0.2580	0.9524	0.9292	0.0611	0.1839	0.1302
TrtXSequence							
VSDH	0	135.00 ± 2.83	4.70 ± 0.14	2.50 ± 1.22	243.00 ± 1.41	19.00 ± 1.41	91.50 ± 0.71
	25	137.50 ± 0.71	4.75 ± 0.21	2.19 ± 0.44	238.50 ± 12.02	23.50 ± 3.54	90.00 ± 1.41
	50	131.00 ± 4.24	4.80 ± 0.28	2.50 ± 0.23	250.00 ± 9.19	19.00 ± 2.82	85.00 ± 4.95
	75	130.50 ± 9.19	4.20 ± 2.12	2.14 ± 0.52	207.00 ± 8.49	21.50 ± 3.53	90.00 ± 1.41
	100	130.50 ± 3.53	4.50 ± 0.42	2.50 ± 1.12	221.50 ± 3.55	19.50 ± 2.12	90.50 ± 9.19
VSDHRh	0	130.00 ± 19.80	4.80 ± 0.42	2.50 ± 0.33	231.00 ± 1.22	22.00 ± 4.95	100.00 ± 1.41
	25	164.00 ± 35.33	4.30 ± 0.42	2.50 ± 0.09	274.00 ± 23.33	41.00 ± 18.38	99.00 ± 1.55
	50	151.00 ± 24.75	5.10 ± 0.42	2.48 ± 0.14	263.00 ± 5.66	37.00 ± 15.56	100.00 ± 2.83
	75	134.00 ± 24.04	3.30 ± 0.25	2.46 ± 0.30	231.00 ± 7.07	29.00 ± 0.33	99.00 ± 4.24
	100	137.00 ± 12.73	4.70 ± 0.88	2.50 ± 0.16	244.00 ± 4.95	17.00 ± 1.41	98.00 ± 1.41
VSDCO_2_	0	125.00 ± 6.36	4.90 ± 0.71	2.15 ± 0.38	241.00 ± 0.71	15.00 ± 0.41	94.00 ± 5.66
	25	128.50 ± 4.95	4.95 ± 0.35	2.50 ± 0.44	231.50 ± 14.85	21.50 ± 6.36	88.00 ± 2.83
	50	129.00 ± 16.97	4.90 ± 0.64	2.46 ± 0.23	240.00 ± 12.02	18.00 ± 2.12	70.00 ± 19.09
	75	146.00 ± 16.97	5.70 ± 0.57	2.50 ± 0.73	235.00 ± 14.14	26.50 ± 12.02	87.00 ± 1.41
	100	124.00 ± 5.66	4.70 ± 0.35	2.50 ± 0.29	218.00 ± 21.92	19.00 ± 2.83	88.00 ± 7.07
P-value		0.2111	0.6838	0.9680	0.1736	0.5017	0.3398

^a^ Seq is baseline = 0, 25 is 25% to average TOD, 50 is 50% to average TOD, 75 is 75% to average TOD, and 100 is average TOD.

^b^ Depicts parameters with a power of analysis of >0.80.

^c^ VSDH, ventilation shutdown plus heat; VSDHRh, ventilation shutdown plus heat and relative humidity; VSDCO_2_, ventilation shutdown plus carbon dioxide.

Hemoglobin (Hb g/dL) data were not recorded by the *i*-STAT® diagnostic system for this parameter.

**TABLE 16:** Changes in the blood chemistry of laying hens overtime when depopulated using VSDH (hyperthemic method), VSDHRh (hyperthermic method), or VSDCO_2_.

Trt^b^	Seq^a^ (%)	pH^c^	pCO_2_ (mmHg)	pO_2_ ^c^ (mmHg)	BEecf^c^ (mmol/L)	HCO_3_ (mmol/L)	TCO_2_ (mmol/L)
VSDH		7.37 ± 0.14	40.20 ± 8.58	62.50 ± 9.20	−2.00 ± 3.72	23.35 ± 2.16	24.50 ± 2.08
VSDHRh		7.41 ± 0.14	38.80 ± 9.23	59.29 ± 13.45	−0.50 ± 3.72	24.35 ± 2.56	25.50 ± 2.63
VSDCO_2_		7.42 ± 0.18	36.80 ± 29.96	61.00 ± 10.71	−0.50 ± 4.20	24.15 ± 3.75	25.00 ± 3.91
P-value		0.8255	0.9801	0.8635	0.8453	0.9210	0.9340
Sequence^a^							
0%		7.40 ± 0.05	38.60 ± 3.98	65.61 ± 47.13	−1.00 ± 2.53	23.95 ± 1.93	25.00 ± 2.00
25%		7.41 ± 0.08	40.53 ± 11.10	56.50 ± 5.79	1.67 ± 4.58	25.70 ± 4.18	26.83 ± 4.26
50%		7.51 ± 0.12	30.85 ± 10.39	54.67 ± 6.82	0.33 ± 4.68	23.27 ± 4.09	24.33 ± 4.18
75%		7.52 ± 0.28	36.82 ± 30.74	51.83 ± 8.94	1.67 ± 3.82	24.20 ± 3.20	25.33 ± 3.88
100%		7.48 ± 0.36	42.72 ± 44.25	41.33 ± 11.79	−2.22 ± 6.22	22.46 ± 2.61	23.50 ± 2.12
P-value		0.0796	0.7903	0.5641	0.5895	0.3696	0.3748
TrtXSequence							
VSDH	0	7.37 ± 0.004^bcd^	40.20 ± 2.45^ab^	62.50 ± 2.12	−2.00 ± 3.2^bcde^	23.35 ± 0.21	24.50 ± 0.71
	25	7.50 ± 0.07^abcd^	34.45 ± 0.07^ab^	40.00 ± 11.31	4.00 ± 5.65^ab^	27.10 ± 4.38	28.00 ± 4.24
	50	7.64 ± 0.03^abc^	25.25 ± 2.62^ab^	46.50 ± 6.36	5.00 ± 1.41^a^	25.40 ± 1.70	26.00 ± 1.41
	75	7.73 ± 0.08^a^	19.00 ± 2.55^b^	45.00 ± 4.24	5.50 ± 2.12^a^	25.00 ± 1.13	25.50 ± 0.71
	100	7.68 ± 0.05^ab^	21.30 ± 0.85^ab^	48.00 ± 1.41	5.00 ± 2.83^a^	24.95 ± 2.05	25.50 ± 2.12
VSDHRh	0	7.41 ± 0.08^abcd^	38.80 ± 7.35^ab^	62.50 ± 47.38	−0.50 ± 2.1^abcd^	24.35 ± 0.35	25.50 ± 0.71
	25	7.42 ± 0.02^abcd^	33.00 ± 3.54^ab^	42.00 ± 1.41	−3.00 ± 4.24^cde^	21.45 ± 3.46	22.50 ± 3.54
	50	7.50 ± 0.09^abcd^	23.75 ± 3.75^ab^	52.00 ± 7.07	−4.50 ± 2.12^de^	18.50 ± 0.99	19.50 ± 0.71
	75	7.67 ± 0.01^ab^	18.40 ± 2.69^b^	55.00 ± 4.24	1.50 ± 2.12^abcd^	21.40 ± 2.40	22.00 ± 2.83
	100	7.72 ± 0.01^a^	17.95 ± 0.35^b^	53.50 ± 16.26	4.00 ± 0.04^ab^	23.35 ± 0.21	24.00 ± 1.02
VSDCO_2_	0	7.42 ± 0.03^abcd^	36.80 ± 3.68^ab^	61.00 ± 12.73	−0.50 ± 4.9^abcd^	24.15 ± 4.17	25.00 ± 4.24
	25	7.33 ± 0.05^cde^	54.15 ± 6.72^ab^	40.00 ± 5.66	2.50 ± 0.71^abc^	28.55 ± 0.07	30.00 ± 0.04
	50	7.38 ± 0.007^bcd^	43.55 ± 5.73^ab^	46.50 ± 5.66	0.50 ± 3.54^abcd^	25.90 ± 3.39	27.50 ± 3.53
	75	7.18 ± 0.10^de^	73.05 ± 27.79^ab^	45.00 ± 1.41	−2.00 ± 2.8^bcde^	26.20 ± 4.38	28.50 ± 4.95
	100	7.04 ± 0.24^e^	88.90 ± 58.12^a^	48.00 ± 1.41	−9.00 ± 1.34^e^	19.10 ± 4.54	21.00 ± 4.35
P-value		0.0002	0.0235	0.3450	0.0226	0.1634	0.1953

^a^ Seq is baseline = 0, 25 is 25% to average TOD, 50 is 50% to average TOD, 75 is 75% to average TOD, and 100 is average TOD.

^b^ VSDH, ventilation shutdown plus heat; VSDHRh, ventilation shutdown plus heat and relative humidity; VSDCO_2_, ventilation shutdown plus carbon dioxide.

^c^ Depicts parameters with a power of analysis of >0.80.

pCO_2_, partial pressure of CO_2_; pO_2_, partial pressure of O_2_; BEecf, base excess in extracellular fluid; HCO_3_, bicarbonate; TCO_2_, total CO_2_.

**TABLE 17:** Blood chemistry change of laying hens overtime when depopulated using VSDH (hyperthemic method), VSDHRh (hyperthermic method), or VSDCO_2_.

Trt^c^		Na (mmol/L)	K^b^ (mmol/L)	iCa^b^ (mmol/L)	Glucose (mg/dL)	Hct (% PCV)	sO_2_ (%)
VSDH		135.00 ± 5.74	4.70 ± 0.70	2.50 ± 0.24	243.00 ± 19.61	19.00 ± 0.72	91.50 ± 6.78
VSDHRh		116.00 ± 18.56	4.50 ± 0.37	2.27 ± 0.37	231.00 ± 41.81	18.50 ± 7.43	99.00 ± 7.90
VSDCO_2_		120.50 ± 10.50	4.40 ± 1.65	2.23 ± 0.17	241.50 ± 31.67	15.00 ± 5.16	90.00 ± 8.53
P-value		0.3788	0.9195	0.3062	0.8694	0.7564	0.2594
Seq^a^							
0%		123.83 ± 12.92	4.53 ± 0.40	2.33 ± 0.26	238.50 ± 3.02^b^	17.50 ± 1.02	93.50 ± 5.05^a^
25%		134.67 ± 16.84	4.65 ± 0.92	2.45 ± 0.12	270.67 ± 38.27^ab^	21.00 ± 8.92	77.00 ± 8.27^b^
50%		129.67 ± 6.31	4.77 ± 0.59	2.33 ± 0.28	276.33 ± 20.30^ab^	16.50 ± 3.21	89.67 ± 1.70^a^
75%		129.33 ± 18.10	4.48 ± 0.55	2.23 ± 0.43	288.17 ± 28.85^a^	22.83 ± 5.92	90.50 ± 5.68^a^
100%		128.33 ± 6.09	5.77 ± 1.98	2.20 ± 0.40	291.17 ± 40.99^a^	16.83 ± 2.23	91.83 ± 2.97^a^
P-value		0.7567	0.0581	0.3062	0.0152	0.3341	0.0013
TrtXSequence							
VSDH	0	135.00 ± 2.82	4.70 ± 0.14^b^	2.50 ± 0.54^a^	243.00 ± 11.41	19.00 ± 0.49	91.50 ± 0.71
	25	138.50 ± 2.12	5.15 ± 1.63^b^	2.50 ± 2.10^a^	265.50 ± 23.33	16.50 ± 2.12	80.00 ± 12.72
	50	129.00 ± 4.24	4.65 ± 0.78^b^	2.50 ± 1.33^a^	277.00 ± 18.38	15.50 ± 0.71	91.00 ± 2.83
	75	126.50 ± 7.77	4.70 ± 0.42^b^	2.50 ± 1.68^a^	255.50 ± 10.61	18.00 ± 4.24	92.00 ± 4.24
	100	131.50 ± 4.95	4.65 ± 0.78^b^	2.50 ± 2.25^a^	248.00 ± 31.11	17.00 ± 2.83	92.50 ± 0.71
VSDHRh	0	116.00 ± 19.79	4.50 ± 0.42^b^	2.27 ± 0.33^abc^	231.00 ± 32.41	18.50 ± 4.95	99.00 ± 1.41
	25	139.00 ± 33.94	4.65 ± 0.78^b^	2.35 ± 0.21^ab^	303.50 ± 54.44	26.00 ± 15.56	78.50 ± 2.12
	50	128.00 ± 12.73	4.45 ± 0.07^b^	2.00 ± 0.19^abc^	280.00 ± 38.18	19.00 ± 3.53	89.50 ± 6.36
	75	117.00 ± 24.04	4.00 ± 1.02^b^	1.70 ± 0.28^bc^	303.50 ± 9.19	27.00 ± 4.58	95.00 ± 1.41
	100	126.50 ± 10.61	4.45 ± 0.07^b^	1.60 ± 0.23^c^	321.00 ± 38.18	17.50 ± 5.65	94.00 ± 4.24
VSDCO_2_	0	120.50 ± 6.36	4.40 ± 0.71^b^	2.23 ± 0.38^abc^	241.50 ± 0.71	15.00 ± 1.21	90.00 ± 5.66
	25	126.50 ± 7.77	4.15 ± 0.07^b^	2.50 ± 1.23^a^	243.00 ± 8.49	20.50 ± 7.78	72.50 ± 10.61
	50	132.00 ± 1.41	5.20 ± 0.71^ab^	2.50 ± 2.11^a^	272.00 ± 14.14	15.00 ± 2.49	88.50 ± 3.53
	75	144.50 ± 14.85	4.75 ± 0.78^b^	2.50 ± 1.12^a^	305.50 ± 27.58	23.50 ± 7.78	84.50 ± 4.95
	100	127.00 ± 4.24	8.20 ± 1.13^a^	2.50 ± 2.55^a^	304.50 ± 10.61	16.00 ± 1.41	89.00 ± 3.78
P-value		0.7712	0.0182	0.0015	0.1633	0.9152	0.9165

^a^ Seq is baseline = 0, 25 is 25% to average TOD, 50 is 50% to average TOD, 75 is 75% to average TOD, and 100 is average TOD.

^b^ Depicts parameters with a power of analysis of >0.80.

^c^ VSDH, ventilation shutdown plus heat; VSDHRh, ventilation shutdown plus heat and relative humidity; VSDCO_2_, ventilation shutdown plus carbon dioxide.

Hemoglobin (Hb g/dL) data were not recorded by the *i*-STAT® diagnostic system for this parameter.

This study was conducted to try to get an understanding of what birds are experiencing throughout different depopulation methods. Due to the nature of this study, the smallest possible sample size that could get approved was utilized. This was to try to get a better understanding of the blood physiology, behaviors, and TOD for laying hens undergoing these treatments. Bird variability could have played a large factor in significance and non-significance. A larger sample size is necessary to allow for a stronger understanding of what exactly is occurring in the laying hen. It would also be beneficial to conduct this study analyzing different parameters to fully understand the stress that the laying hens are undergoing.

## 5 Conclusion

As HPAI continues to be a major concern for the poultry industry, it is important to know the methods used for emergency depopulation and how they affect the laying hen. This study examined how three treatments, VSDH, VSDHRh, and VSDCO_2,_ were compared with respect to laying hen TOD, blood physiology, and behaviors. VSDCO_2_ had a significantly shorter TOD than the other two treatments. While the VSDH and VSDHRh treatments were not significantly different, VSDHRh had a time reduction of 16%. There also were no significant differences associated with the hens’ EEGs nor their behaviors. There were significantly greater levels of HSP70 production in VSDCO_2_ compared to the other two treatments. Overall, this study demonstrated that while VSDCO_2_ may have significantly quicker TOD, in the event that there are limited supplies, both VSDH and VSDHRh may be available as alternatives to depopulate laying hen houses. Having multiple alternative methods that have met the guidelines as defined in the Red Book (USDA-APHIS, 2017), like VSDH and VSDHRh, may allow for the reduction in the spread of HPAI. The approval for use comes from the AVMA Depopulation Guidelines; however, the USDA makes the final decision on which methods will be administered depending on different factors such as bird type and housing type. Future research with these treatments may potentially provide a better understanding of laying hen stress and could measure respiratory rate, internal body temperature measurements, thyroid hormones like T3 and T4, and adrenocorticotropic hormone (ACTH) levels.

## Data Availability

The original contributions presented in the study are included in the article/Supplementary Material; further inquiries can be directed to the corresponding author.

## Abbreviations

HPAI, highly pathogenic avian influenza; VSD+, ventilation shutdown plus; VSDH, ventilation shutdown plus heat; VSDHRh, ventilation shutdown plus relative humidity; VSDCO_2_ ventilation shutdown plus carbon dioxide; CO2, carbon dioxide; mV, millivolts; EEG, electroencephalographic; Hz, hertz; TOD, time of death; HSP70, heat shock protein 70; Rh, relative humidity; min, minutes.
